# Efficacy of intravitreal ziv-aflibercept in patients with macular edema following retinal vein occlusion in Korle-Bu Teaching Hospital, Ghana: a retrospective case series

**DOI:** 10.1007/s10792-021-01799-w

**Published:** 2021-03-29

**Authors:** Imoro Zeba Braimah, Kofi Agyabeng, Winfried M. Amoaku

**Affiliations:** 1 Department of Surgery (Eye), University of Ghana Medical School, Korle Bu, Accra, Ghana; 2 Lions International Eye Centre, Korle- Bu Teaching Hospital. Korle-Bu, Accra, Ghana; 3 Department of Mathematics, KU Leuven, Leuven, Belgium; 4 Academic Ophthalmology, DCN, Faculty of Medicine and Health Sciences, University of Nottingham, Nottingham, UK

**Keywords:** Optical coherence tomography, Cystoid macular edema, Branch retinal vein occlusion, Central retinal vein occlusion, Hemi-retinal vein occlusion

## Abstract

**Aim:**

To evaluate the efficacy of ziv-aflibercept in Ghanaian patients with macular edema (ME) secondary to retinal vein occlusion (RVO).

**Methodology:**

In this retrospective study, the medical records of patients with ME secondary to RVO who had been treated with intravitreal ziv-aflibercept (IVZ) (1.25 mg/0.05 ml), as part of routine clinical practice, on pro re nata basis with a minimum follow-up of 6 months were retrieved and analyzed. The main outcome measures are mean change in best-corrected visual acuity (BCVA) and central subfield foveal thickness (CSFT) measured on optical coherence tomography from baseline to 12 months post-IVZ, and ocular and systemic safety.

**Results:**

Forty-three eyes were included in this study. Their mean age was 62.8 ± 11.9 years, 67.4% had at least 12-month duration of follow-up, 50% had primary open-angle glaucoma and 38 (88.4%) eyes were treatment naive. There was significant improvement in mean BCVA in LogMAR at 1 month post-initiation of IVZ (0.8 ± 0.5 vs. 1.1 ± 0.6), and visual improvement was maintained up to 12 months (*p* < 0.001). Eyes with ME following BRVO had better mean BCVA at baseline and on subsequent visits compared to eyes with CRVO/HRVO (*p* = 0.01). There was significant reduction in mean CSFT up to 12 months post-IVZ injection compared to baseline (*p* < 0.001). Ocular complications observed were consistent with complications associated with RVO.

**Conclusion:**

We have observed significant improvement in functional and anatomic outcomes 12 months post-initiation of IVZ. There is the need to confirm long-term efficacy and safety of IVZ in a large prospective study.

## Introduction

Retinal vein occlusion (RVO) is acquired retinal vascular disorder and is the second commonest cause of blindness from retinal vascular diseases after diabetic retinopathy [1]. The prevalence of RVO is 5.2 per 1000 persons [2] and is classified into branch RVO (BRVO), central RVO (CRVO) and hemi-retinal vein occlusion (HRVO). The prevalence of BRVO and CRVO is 4.4 and 0.8 per 1000 persons, respectively [2]. Macular edema (ME) is the most common cause of visual loss in RVO and is associated with decreased vision-related quality of life [3, 4].

Vascular endothelial growth factor (VEGF) plays an important role in the pathogenesis of ME following RVO [5, 6]. VEGF promotes vascular leakage, resulting from increasing the permeability of retinal vessels [5, 6]. The use of anti-VEGF agents has been shown to improve vision or prevent severe visual loss in patients with ME following RVO [7, 8].

The CRUISE, COPERNICUS, and GALILEO trials and report from Epstein et al. have shown that ranibizumab, aflibercept, and bevacizumab were safe and effective in the treatment of ME from RVO [9–12]. Rajagopal et al. reported that bevacizumab and ranibizumab had similar efficacy at 6 months in patients with RVO in the CRAVE study [13].

There are few reports on the safety and efficacy of off-label intravitreal ziv-aflibercept (IVZ) in patients with ME secondary RVO [14–20]. However, no reports exist on the efficacy of IVZ in African patients with macular edema secondary to RVO. In this retrospective study, we seek to evaluate the efficacy of IVZ 1.25 mg/0.05 ml in Ghanaian patients with ME associated with RVO.

## Methods

This is a retrospective, observational case series of patients with ME secondary to RVO who had been treated with IVZ from October 2016 to March 2018 at the Korle-Bu Teaching Hospital (KBTH) in Ghana. The protocol was approved by the Ethical and Protocol Review Committee of the College of Health Sciences, University of Ghana (CHS-Et/M.6-P1.1/2017-2018), and the study adhered to the tenets of the Declaration of Helsinki.

All patients with ME from RVO who had consented to treatment with off-label IVZ at the KBTH were included in this study. A diagnosis of RVO was made when the history and clinical examination findings were consistent with RVO. These included dilatation and tortuosity of one or more retinal veins, dot/blot and flame-shaped hemorrhages involving one or more quadrants with or without exudates or cotton wool spots or optic disc edema; other causes of similar fundus picture were excluded. Patients were included if they were aged 18 years or older, met diagnostic criteria for RVO, were treatment-naïve or had not received any treatment in the preceding 3 months, had center-involving ME with retinal thickness > 300um on spectral-domain optical coherence tomography (SD-OCT), and had a minimum follow-up of 6 months post-initiation of IVZ. Eyes with ME secondary to RVO were excluded from the study if they had intraocular surgery in the study eye within 3 months, or laser photocoagulation or intravitreal corticosteroid within 3 months of IVZ, or myopia ≥ − 6.0 diopters. Re-treatment with IVZ was based on clinical need as determined by the treating physician.

The clinical records of eligible patients were retrieved from the medical records department of the Eye Centre, Korle-Bu Teaching Hospital by the principal investigator. The clinical characteristics recorded included: age, sex, ethnicity, systemic co-morbidities, affected eye, type, number and duration of previous anti-VEGF injections at baseline. Best-corrected visual acuity (BCVA), central subfield foveal thickness (CSFT), presence of intraretinal fluid (IRF) or subretinal fluid (SRF) at baseline and at each visit whilst on IVZ therapy were recorded. Intraocular pressure (IOP) measurements at each visit and the number of IVZ and additional treatment whilst on IVZ, if any, were also recorded.

BCVA was assessed by Early Treatment Diabetic Retinopathy Study (ETDRS) R chart (Precision Vision, La Salle, Illinois, USA). CSFT is the mean thickness in the central 1000-µm-diameter area (innermost ETDRS circle) measured on Oct acquired with the three-dimensional OCT-2000 Topcon (Topcon, Tokyo, Japan).

Disease activity recurrence was defined as a macula that is dry (absence of IRF and SRF) followed by the observation of fluid at subsequent visits, BCVA loss of at least one line on the VA chart with evidence of fluid in the macula or increase in CSFT of at least 100 µm on OCT.

### IVZ

IVZ was administered as per institutional protocol. Details of the protocol for the preparation of IVZ prior to intravitreal injection have been reported previously [19]. The intravitreal injection is done using a sterile technique. Topical anesthetic agent proparacaine and 5% povidone iodine were instilled into the conjunctival cul-de sac and periocular skin, eyelids, and lashes cleaned using 10% povidone iodine. The eye was draped and the injection (ziv-aflibercept, 1.25 mg/0.05 ml) given into the mid-vitreous cavity 4 mm or 3.5 mm posterior to the limbus in phakic and pseudophakic eyes, respectively. Hand motion vision was checked and confirmed to be present at the end of the procedure. No topical antibiotics were given prior to, during or after each injection.

### Outcome measures

The primary outcome of interest was the mean change in BCVA in LogMAR (ETDRS) letters from baseline to 12 months post-initiation of IVZ. Secondary outcome measures were the proportion of eyes that gained ≥ 10 letters from baseline, the proportion of eyes that gained ≥ 15 letters from baseline, a change in CSFT on OCT from baseline to 12 months, ocular adverse events including incidence of non-infectious intraocular inflammation, endophthalmitis, and systemic events, whether drug-related or unrelated.

### Statistical analysis

STATA software V14.2 (StataCorp, College Station, TX, USA) was used for statistical analyses. Continuous variables were presented as mean and standard deviation, while frequencies were reported for categorical variables. Categorical variables were compared using Chi-square or Fisher’s exact test of association. Baseline as well as final visit information on age, BCVA, IOP, number of injections and CSFT levels were compared between treatment types using the Welch’s t-test for comparing means. Repeated-measures ANOVA with nesting test was used in comparing variations in BCVA, IOP, CSFT, number of recurrence, and number of injections by RVO type (BRVO vs. CRVO/HRVO), over time as well as interaction between RVO type and time at month 0, 1, 3, 6, 9, and 12. A graphical presentation of the changes in BCVA, IOP, CSFT and number of recurrence over the observed duration were also done. Welch t-test was used to compare BCVA, IOP, CSFT, number of recurrence, and number of injections by RVO type at baseline and final visits. Paired t-test was also employed to compare BCVA, IOP, CSFT, number of recurrence, and number of injections at each follow-up time to baseline measurements as confirmatory analysis in support of the repeated-measures ANOVA with nesting test. Statistical level of significance was set at 5%.

## Results

Forty-three eyes of 42 patients (24 females) who had been treated with IVZ with a minimum follow-up of 6 months were included in this study. The average ± standard deviation (range) age was 62.8 ± 11.9 (34–86) years. They were followed for an average ± standard deviation (range) duration of 11.8 ± 4.1 (6–17) months, and 29 (67.4%) eyes had follow-up duration of at least 12 months. Twenty-four eyes had BRVO, 16 CRVO, and 3 HRVO. Twenty-one (50%) patients had primary open-angle glaucoma. Five eyes had received previous injections of bevacizumab prior to IVZ, with the mean number of previous anti-VEGF injections of 3.8 ± 3.6 (1–10), median 2. The baseline clinical characteristics of the participants are summarized in Table 1. The mean number of IVZ injections at 3, 6, 9, and 12 months post-initiation of IVZ is shown in Table 2.

**Table 1:** Baseline characteristics of patients with macular edema secondary to RVO in KBTH

Parameter	Total (*n* = 43)	Treatment		Chi-square
		BRVO (*n* = 24)	CRVO/HRVO (*n* = 19)	*P* value
Age in complete years: mean ± SD	62.8 ± 11.9	61.0 ± 14.2	65.1 ± 7.8	0.242 **§**
Sex: male/female	19/24	11/13	8/11	0.807
Glaucoma: yes/no	22/21	10/14	12/7	0.161
Systemic hypertension: yes/no	39/4	23/1	16/3	0.306®
Diabetes mellitus: yes/no	11/32	6/18	5/14	0.921
Hyperlipidemia: yes/no	3/40	2/22	1/18	1.000®
Previous Treatment: yes/no	5/38	2/22	3/16	0.640®
IRF: yes/no	43/0	24/0	19/0	–
SRF: yes/no	28/15	14/10	14/5	0.294
BCVA: mean ± SD	1.1 ± 0.6	0.9 ± 0.5	1.5 ± 0.5	**< 0.001 §**
IOP in mmHg: mean ± SD	17.5 ± 4.2	16.5 ± 3.7	18.8 ± 4.5	0.077 **§**
CSFT in μm: mean ± SD	502.8 ± 155.3	498.6 ± 135.0	508.1 ± 181.5	0.850 **§**

*BCVA* best-corrected visual acuity in LogMAR, *CSFT* central subfield fovea thickness, *IOP* intraocular pressure, *n* number, *SD* standard deviation

® = Fischer’s exact test, § = *P* value from Welch’s *t* test for comparing means

Bold indicates stat (*p* values) considered to be significant

**Table 2:** Comparison of changes in BCVA, IOP, CSFT and number of recurrence between types of RVO over a 12-month duration using repeated-measures ANOVA with nesting

Variable	BRVO (*n* = 24)	CRVO/HRVO (*n* = 19)	Overall (*n* = 43)	*P* value*	*P* value**	*P* value***
	Mean ± SD	Mean ± SD	Mean ± SD			
*BCVA*						
Baseline	0.9 ± 0.5	1.5 ± 0.5	1.1 ± 0.6	**0.010**	**< 0.001**	0.118
1 Month	0.6 ± 0.4	1 ± 0.6	0.8 ± 0.5			
3 Months	0.5 ± 0.4	0.9 ± 0.6	0.7 ± 0.5			
6 Months	0.5 ± 0.5	0.9 ± 0.6	0.7 ± 0.6			
9 Months (*n* = 29)	0.6 ± 0.4	0.8 ± 0.6	0.7 ± 0.5			
12 Months (*n* = 29)	0.6 ± 0.5	0.8 ± 0.6	0.7 ± 0.5			
Final visit (*n* = 43)	0.6 ± 0.5	0.8 ± 0.6	0.7 ± 0.5	0.178§		
*IOP*						
Baseline	16.5 ± 3.7	18.8 ± 4.5	17.5 ± 4.2	**0.037**	0.714	0.837
1 Month	16.2 ± 4.2	18.4 ± 5.3	17.2 ± 4.8			
3 Months	16.5 ± 3.3	19.3 ± 4.3	17.7 ± 4.0			
6 Months	16.1 ± 2.8	18.7 ± 4.9	17.2 ± 4.0			
9 Months (*n* = 29)	17.2 ± 3.6	19.6 ± 6.7	18.2 ± 5.2			
12 Months (*n* = 29)	15.7 ± 3.9	18.8 ± 10.4	17.1 ± 7.6			
Final visit (*n* = 43)	17.4 ± 8.3	17.9 ± 5.6	17.7 ± 7.1	0.804§		
*CSFT*						
Baseline	498.6 ± 135	508.1 ± 181.5	502.8 ± 155.3	0.882	**< 0.001**	0.385
1 Month	248 ± 74.6	265.8 ± 81.4	255.9 ± 77.2			
3 Months	226.9 ± 79.9	241.9 ± 74.4	233.5 ± 77			
6 Months	236.2 ± 67	258.5 ± 108.2	246 ± 87.2			
9 Months (*n* = 29)	275.7 ± 119.3	308.4 ± 142.6	289 ± 127.7			
12 Months (*n* = 29)	339.9 ± 200.6	253.4 ± 58.6	301.5 ± 158.3			
Final visit	273.3 ± 129.0	246.1 ± 72.5	261.6 ± 108.2	0.393§		
*Number of recurrence*						
6 Months (*n* = 43)	0.7 ± 0.6	0.5 ± 0.5	0.6 ± 0.6	0.9537	**< 0.001**	0.184
9 Months (*n* = 21)	1.1 ± 0.7	1.3 ± 1	1.1 ± 0.8			
12 Months (*n* = 24)	1.3 ± 0.5	1.7 ± 0.9	1.4 ± 0.7			
*Number of injections*						
3 Months (*n* = 43)	2.5 ± 0.6	2.4 ± 0.5	2.5 ± 0.5	0.801	**< 0.001**	0.615
6 Months (*n* = 43)	3.3 ± 1.0	3.3 ± 1.1	3.3 ± 1.0			
9 Months (*n* = 29)	4.1 ± 1.6	4.4 ± 1.7	4.3 ± 1.6			
12 Months (*n* = 29)	4.8 ± 1.8	5.3 ± 2.0	5.0 ± 1.9			
Final visit (*n* = 43)	4.6 ± 2.1	5.2 ± 2.7	4.8 ± 2.4	0.452§		

*BCVA* best-corrected visual acuity, *CSFT* central subfield fovea thickness, *IOP* intraocular pressure, *n* number, *SD* standard deviation

^*^P value: *P* value from ANOVA test for comparing means between treatment type, **P value: *P* value from Huynh–Feldt epsilon for comparison of means over time, ***P value: *P* values from Huynh–Feldt epsilon for comparison of means over treatment type and time (interaction). § *P* value from welch *t* test for comparing means

Bold indicates stat (*p* values) considered to be significant

### Visual outcome

**Fig. 1:**
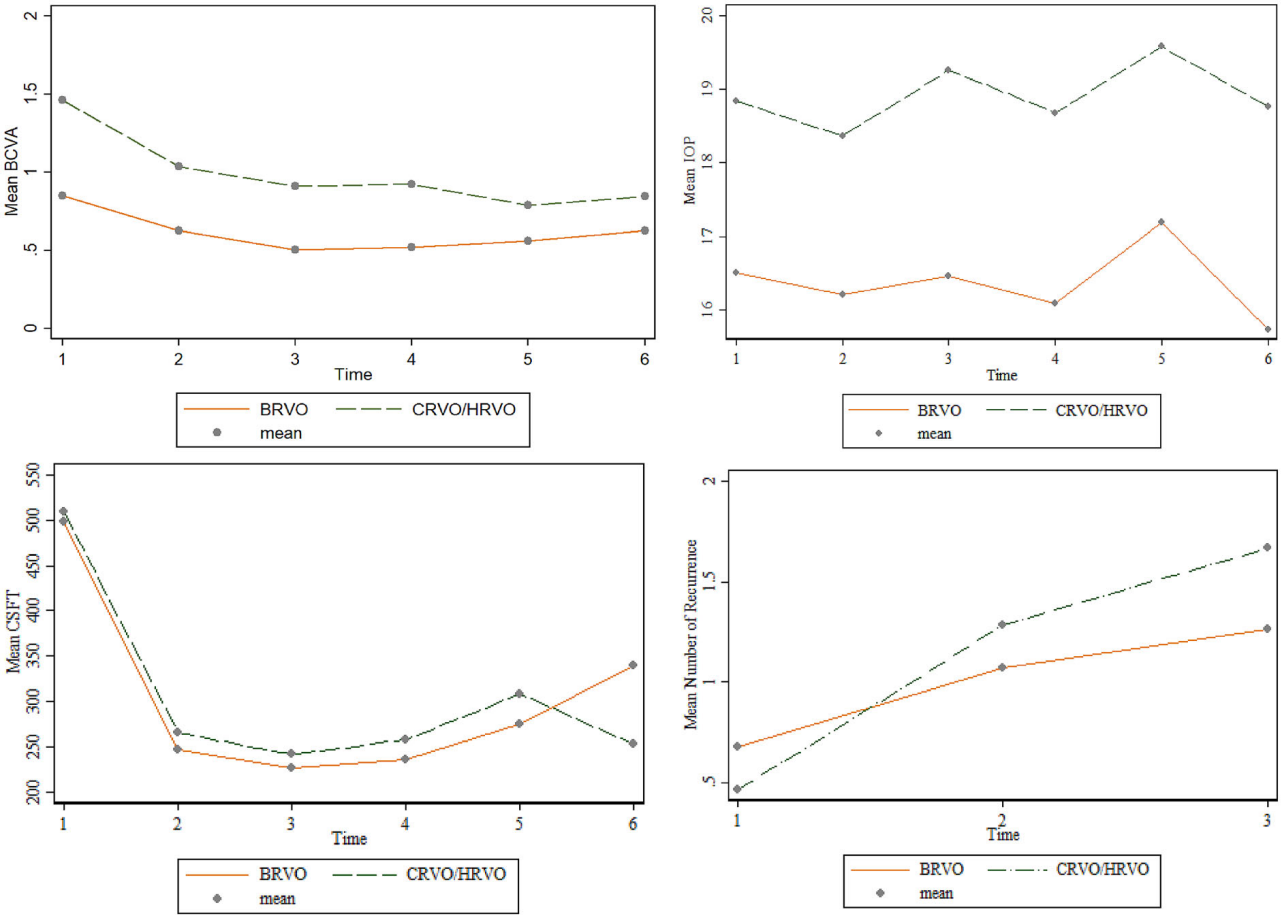
Mean change in clinical parameters over time by RVO type. BCVA = best-corrected visual acuity, BRVO = branch retinal vein occlusion, CRVO/HRVO = central retinal vein occlusion/hemi-retinal vein occlusion. CSFT = central subfield fovea thickness, IOP = intraocular pressure, 1 = baseline, 2 = 1 months post-treatment, 3 = 3 months post-treatment, 4 = 6 months post-treatment, 5 = 9 months post-treatment, 6 = 12 months post-treatment for BCVA, IOP, and CSFT. 1 = 6 months post-treatment, 2 = 9 months post-treatment, 3 = 12 months post-treatment for number of recurrence

There was significant improvement in mean BCVA at 1, 3, 6, and 12 months, respectively, compared to baseline (*p* < 0.001) (Table 2 and Fig. 1). The VA improvement observed at 1 month post-initiation of IVZ was maintained up to 12 months and the last follow-up visit (Table 2). Eyes with BRVO had better mean BCVA at baseline and at 1, 3, 6, 9, and 12 months post-initiation of IVZ compared to eyes with CRVO/HRVO (Fig. 1). Twenty-nine (67.4%) eyes had a visual gain of at least 2 lines, and 25 (58.1%) had a visual gain of at least 3 lines at 6 months post-initiation of IVZ. Also, 20 (70%) of 29 eyes had a VA gain of at least 2 lines, and 18 (62.1%) had a VA gain of at least 3 lines at 12 months. Of the 5 eyes treated with bevacizumab prior to IVZ, 2 had visual gain of at least 1 line, 2 maintained their vision and 1 had vision decline despite having a dry macular at 6 months post-initiation of IVZ. There was no significant difference in the mean LogMAR acuity between eyes, which had recurrence of retinal fluid compared to eyes which had no recurrence, at the last follow-up visit (0.7 ± 0.4 vs. 0.6 ± 0.7, *p* = 0.763). One eye with ischemic CRVO, which developed neovascular glaucoma, had no perception of light at the last follow-up visit despite treatment with hypotensive medications and diode laser transscleral cyclophotocoagulation.

### Anatomic outcome

There was significant reduction in the CSFT at 1, 3, 6, 9, and 12 months compared to baseline (*p* < 0.001) (Table 2 and Fig. 1). There was no significant difference in the CSFT between eyes with BRVO compared to CRVO/HRVO at baseline and at 1-, 3-, 6-, 9-, and 12-month visits. Six (13.9%) eyes had persistent IRF at 6 months and 9 months post-initiation of IVZ. Thirteen (30.2%) eyes had no recurrence of fluid, whilst 25 (58.1%) had at least one recurrence at the last follow-up visit. Eyes with recurrence of retinal fluid had significantly more injections of IVZ compared to eyes who had no recurrence at the last follow-up visit (5.2 ± 1.9 vs. 3.3 ± 1.1, *p* = 0.004). Similarly, eyes with recurrence of retinal fluid had significantly longer follow-up duration than eyes that had no recurrence (13.3 ± 3.3 vs. 7.6 ± 2.3, *p* < 0.001).

### Adverse events

**Fig. 2:**
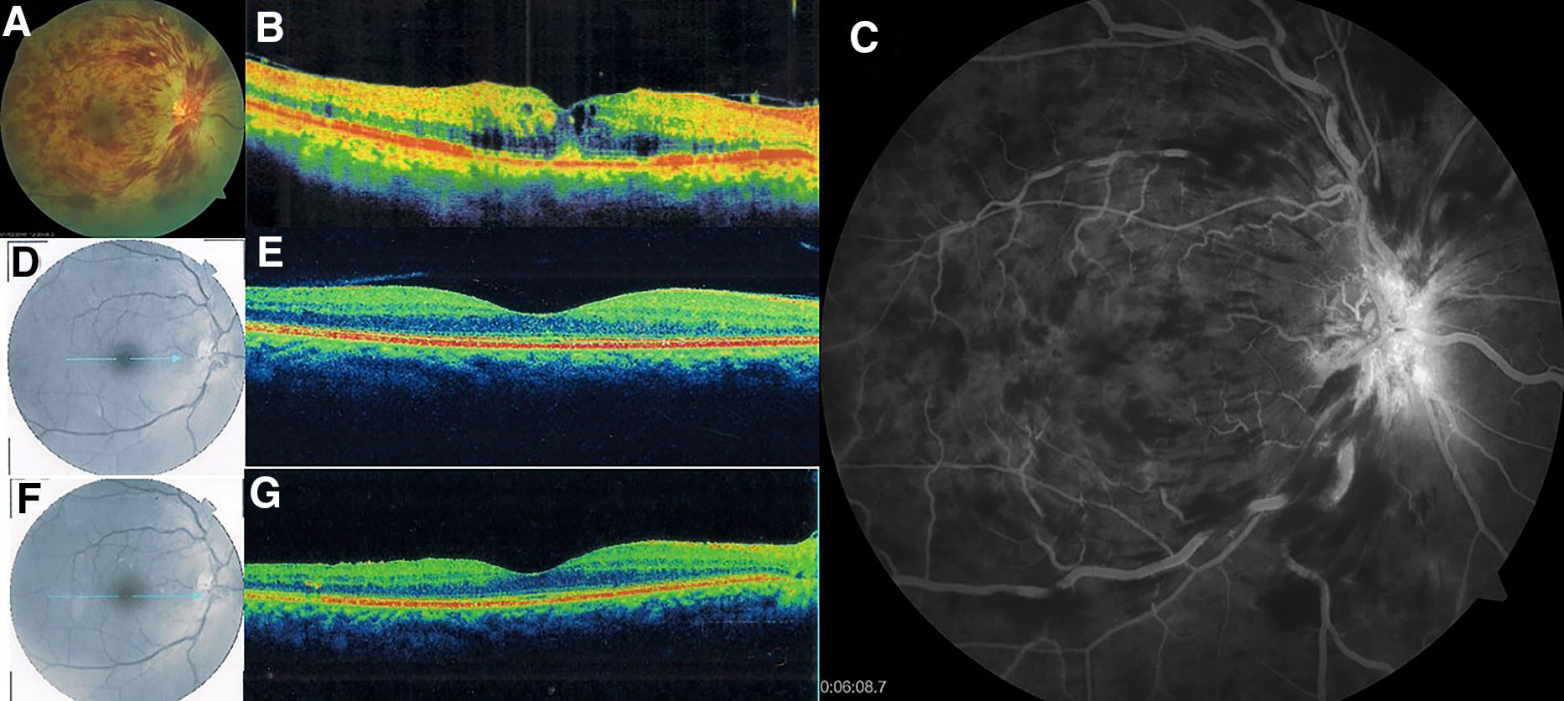
Fundus photograph (**a**), fluorescein angiograph (**c**), and SD-OCT b-scans (**b**, **e** and **g**) of a 59-year-old female presenting with central retinal vein occlusion in the right eye. Status of macular 12 weeks after initiation of ziv-aflibercept 1.25 mg/0.05 ml injection (**d**, **e**). Presence of epiretinal membrane 17 months after initiation of intravitreal ziv-aflibercept (**f**, **g**)

A total of 383 injections were given during the study period. Twelve (25.6%) of the eyes had at least one ocular complication during the study period. Of these, 5 had epiretinal membranes, 1 vitreomacular traction syndrome, 2 eyes developed raised IOP, another 2 developed neovascular glaucoma, 1 developed lamellar hole and 1 developed neovascularization elsewhere on the retina associated with vitreous hemorrhage, and 1 eye developed crystalline retinopathy. No eye developed drug-related adverse events such as intraocular inflammation and endophthalmitis post-injection. Figure 2 shows the images of a patient presenting with CRVO who was noted to have epiretinal membrane at the last follow-up visit.

## Discussion

This study reports a significant improvement in BCVA and a significant reduction in CMT following IVZ of 1.25 mg in routine clinical practice in a Ghanaian population. The improvement in BCVA and reduction in CMT observed at 1 month was maintained up to 12 months post-initiation of IVZ.

Our findings of significant gain in vision in eyes with BRVO and CRVO at 1 year following administration of IVZ in real-world setting are consistent with other studies from other populations [15–17]. Chan et al. [16] observed significant visual improvement at 1 year in eyes with BRVO that received IVZ. Eldeeb et al. [17] in a series of 6 eyes of 6 patients with ME following CRVO treated with 1.25 mg IVZ on a pro re nata (prn) basis achieved significant mean VA gain at 12 months compared to baseline. Other studies on anti-VEGF agents such as bevacizumab, ranibizumab, and aflibercept have reported similar visual gains [9–13, 21]. The visual gain of at least 3 lines in 62% of eyes at 1 year in our series is also similar to other studies on RVO [11, 12] but superior to those reported by Spooner et al. using bevacizumab, ranibizumab, or aflibercept in a Caucasian population [21]. Long-term visual outcomes following intravitreal anti-VEGF therapy have been reported by Spooner et al. and Maggio et al. [21, 22]. The retrospective study of Spooner et al. [21] included 68 eyes with RVO; observed visual gains at 1 year were maintained at 5 years. Maggio et al. [22] also found significant visual gain at 4 years following flexible dosing regimen of ranibizumab and dexamethasone in eyes with ME associated with RVO. Our finding of significantly better BCVA in the BRVO group compared to the CRVO/HRVO group in all visits is supported by other retrospective studies in real-world clinical settings [21, 22].

Significant reduction in CSFT in eyes with RVO following administration of IVZ on prn bases has been reported similar to this series [15–17]. These observations are further supported by other studies on outcome of ME associated with RVO-treated other anti-VEGFS [9–13, 21–23]. In our study, baseline CSFT and mean reduction in reduction in CFST were not significantly different between BRVO and CRVO, although VA was significantly better in the BRVO compared to CRVO group in all visits. In the retrospective study by Spooner et al. [21], the mean CMT at baseline was significantly worse in the CRVO compared to BRVO group although no difference was noticed at 1 year. In a study by Maggio et al. [22], the mean CMT was significantly lower in BRVO eyes at baseline and at year 1 although no such differences were found in subsequent years up until year 5. Such differences may be related to the different parameters used (CSFT vs. CMT), or the duration of ME.

The intravitreal use of ziv-aflibercept (1000 mOsm/Kg) may be potentially toxic to the retina due to its hyperosmolality. However, Chabblani et al. and Mansour et al. did not observe clinical toxicity following IVZ therapy [24, 25]. The ocular adverse events observed in a large retrospective study of 5914 IVZ were consistent with the safety profile of aflibercept and other anti-VEGF agents [18]. Further safety profile was provided by a retrospective study of eyes who received at least 10 IVZ injections [20]. Paulose et al. [15] observed anterior chamber flare in one eye following IVZ injection in 9 eyes with RVO. Eldeeb et al. and Chan et al. did not observe ocular or systemic adverse events following IVZ in eyes with RVO [16, 17]. The ocular adverse events noted in this series can be attributed to recognized complications of retinal vein occlusions and/or associated ME [21, 22, 26].

Our study has inherent limitations including the retrospective design, small sample size, missing data for some visits due to real-life clinical settings, absence of differentiation of ischemic from ischemic RVO, and no angiographic analysis. However, despite these limitations, this study provides efficacy data of IVZ in a Ghanaian African populations and adds to literature on efficacy of IVZ in other populations. The low cost of IVZ makes an attractive alternative to aflibercept particularly in developing and low–middle-income countries who have to pay out of pocket for intravitreal injections.

## Conclusion

IVZ use in routine clinical practice on pro re nata basis was associated with significant improvement in visual and anatomic outcomes. Ocular adverse events including occurrence of endophthalmitis or systemic complications were not observed during the study period. Prospective randomized clinical trials are needed to validate the efficacy of IVZ in the treatment of ME secondary to RVO.
